# Social Sustainability in Late Adolescence: Trait Emotional Intelligence Mediates the Impact of the Dark Triad on Altruism and Equity

**DOI:** 10.3389/fpsyg.2022.840113

**Published:** 2022-02-15

**Authors:** Marco Giancola, Massimiliano Palmiero, Simonetta D’Amico

**Affiliations:** Department of Biotechnological and Applied Clinical Sciences, University of L’Aquila, L’Aquila, Italy

**Keywords:** late adolescence, personality, dark triad, trait emotional intelligence, pro-social sustainability, altruism, equity

## Abstract

Adolescence involves a profound number of changes in all domains of development. Among others, adolescence yields an enhanced awareness and responsibility toward the community, representing a critical age to develop prosocial behaviors. In this study, the mediation role of Trait Emotional Intelligence (TEI) was detected for the relationship between the dark triad and prosocial behavior based on altruism and equity. A total of 129 healthy late adolescents filled in the Dark Triad Dirty Dozen, measuring Machiavellianism, psychopathy, and narcissism; the Altruistic Action Scale, evaluating behaviors directed at helping others; the Equity Scale, assessing behaviors directed at equity in different forms; and the TEI Questionnaire-Short Form. Results showed that TEI mediated the negative effects of the three dark triad traits on both altruism and equity. This finding suggests that TEI, which relies on a set of dispositions (e.g., emotional management of others, social competence, and empathy), might reduce the malevolent effects of the dark triad on altruism and equitable behavior in late adolescence. This led to assume that intervention programs focused on improving emotional skills, also in late adolescence, can promote prosociality.

## Introduction

Exploring what brings people to engage in prosocial actions is relevant for targeting the social crisis of our times. Specifically, late adolescence (18–22 years) depicts the passage to adulthood characterized by an enhanced civic involvement and responsibility toward the community that brings to a deeper understanding of the social and cultural settings in which people live (Zarrett and Eccles, 2006). Tackling the social crisis implies moving to a set of deliberate, effective, and anticipatory actions focused on safeguarding the social environment, ensuring the social sustainability of the planet for today and forthcoming generations. According to Tapia-Fonllem et al. (2013) framework, social sustainability lies in the concept of inclusiveness by embracing prosocial practices such as altruistic and equitable actions (Tapia-Fonllem et al., 2013). Altruism reflects a set of actions that benefit others at a personal cost (Kerr et al., 2004), while equity relies on actions guaranteeing a fair distribution of natural and social resources among people (Corral-Verdugo et al., 2015). Although people vary in expressing their prosociality in daily life, personality represents one of the main contributors to such individual differences (Oda and Matsumoto-Oda, 2022). We considered the personality-prosocial behavior association in late adolescence, focusing on the understated role of the dark triad on social sustainability through the effect of Trait Emotional Intelligence (TEI).

### The Dark Triad and Prosocial Behaviors

The dark triad (Machiavellianism, psychopathy, and narcissism; Paulhus and Williams, 2002) depicts a constellation of subclinical and malevolent personality traits, sharing a common core in terms of antisocial and aversive behaviors, including a tendency to deceive, manipulate, and exploit others (Paulhus and Williams, 2002). Machiavellianism lays on cynical behaviors and the inability to recognize the emotions of others (Láng and Birkás, 2014). In turn, psychopathy relies on callous affect, erratic lifestyle, and antisocial behavior (Neumann et al., 2007), whereas narcissism incorporates a blend of vanity, egocentric admiration, and a desire for superiority that negatively impacts social relationships (Kauten and Barry, 2016). Overall, adolescents with high dark triad tend to be antisocial, vulgar, and academically disengaged (Zhang et al., 2015). Specifically, adolescents with high Machiavellianism show emotional problems such as lack of sympathy (Sutton and Keogh, 2000) and behavioral problems (Zhu et al., 2021), including aggression, a higher tendency toward antisocial practices, lower altruistic concerns (Swami et al., 2010), and equity sensitivity (Woodley and Allen, 2014). Psychopathy is also negatively related to prosocial practices (Papageorgiou et al., 2020) and different social skills such as communication, group collaboration (Anwar and Zubair, 2021), altruistic prosociality (White, 2014), and equity sensitivity (Woodley and Allen, 2014). Regarding narcissism, research emphasized a positive association (Papageorgiou et al., 2020), revealing that to increase the admiration of others, narcissists intentionally distort the memory of events to increment their self-esteem (Hart et al., 2018), and, in turn, accommodate their grandiose image as altruistic people (Palmer and Tackett, 2018). However, some studies revealed that this trait was positively related to antisocial and aggressive behaviors and, thus, negatively associated with prosociality: through aggressive behaviors, narcissists can dominate over peers and reinforce their grandiose self-image (Fanti and Henrich, 2015). Notably, narcissism was also found uncorrelated to prosocial behavior, such as equity sensitivity (Woodley and Allen, 2014).

### Dark Triad and Trait Emotional Intelligence

Trait Emotional Intelligence involves a constellation of emotion-related dispositions, which lay at the lower levels of personality hierarchies (Petrides and Furnham, 2001; Petrides et al., 2007). Research about the relationships between the dark triad and TEI in adolescents is extremely scarce. Zhang et al. (2015) revealed that adolescents (13–19 years old) with higher levels of Machiavellianism and psychopathy showed lower levels of TEI, whereas adolescents with higher levels of narcissism demonstrated higher levels of TEI. Studies on adults showed that both psychopathy (Petrides et al., 2011; Jauk et al., 2016) and Machiavellianism (Austin et al., 2014) were negatively related to TEI (Miao et al., 2019), although Machiavellianism was also found positively related to higher levels of TEI in men (Jauk et al., 2016) and the perspective-taking facet of cognitive empathy (Szabó and Bereczkei, 2017). The positive Machiavellianism-TEI association can rely on emotional competence as a necessary means for manipulative behavior (Jauk et al., 2016). Yet, a meta-analytic review of Miao et al. (2019) showed that narcissism and TEI are unrelated, since some studies showed positive correlations between narcissism and the different facets of TEI (Petrides et al., 2011; Szabó and Bereczkei, 2017), including assertiveness (Watson et al., 1988), optimism (Farwell and Wohlwend-Lloyd, 1998), positive social relationships (Foster and Campbell, 2005), whereas others showed negative relationships, as narcissists showed lower levels of empathic concern (Watson and Biderman, 1994). These contradictory results can be in part due to the double facet of narcissism, involving grandiosity (high self-esteem, interpersonal dominance, and tendency to overestimate one’s capabilities), or vulnerability (defensive, avoidant, insecure, hypersensitive, and vigilant for criticism) (Miller et al., 2011). Thus, grandiosity would be positively related to TEI, whereas vulnerability would be negatively related (Zajenkowski et al., 2018).

### Trait Emotional Intelligence and Prosocial Behaviors

Adolescence marks the development of more efficient forms of prosocial behaviors persisting into adulthood (Eisenberg et al., 2005). Adolescents’ development depends on both social contexts and personality characteristics such as TEI (Gallitto and Leth-Steensen, 2019). Emotional and social competencies underpinned by TEI (e.g., emotionality and sociability) are crucial in acting prosocially (Petrides et al., 2006). Managing emotions allows people to elaborate their own perception of people’s feelings into motivation to help and collaborate with others (Zhao et al., 2020). Petrides et al. (2006) found that higher TEI scores were related to more nominations from children for being cooperative, kind, and having leadership qualities and fewer nominations for being a bully. In addition, TEI was positively correlated with social competencies and kindness. Mavroveli et al. (2007) confirmed that adolescents with higher TEI scores were more likely to be nominated as cooperative by their classmates, whereas Mavroveli and Sánchez-Ruiz (2011) showed that higher TEI was associated with higher peer-ratings for prosocial and fewer nominations for antisocial behaviors. In addition, youth emotional and sociability competencies underpinned caring and connections with others positively predicted social sustainability both in terms of altruism and equity (Giancola et al., 2021).

### The Current Study

Based on personality approach on prosociality (e.g., Oda and Matsumoto-Oda, 2022), describing personality as one of the main contributors to people’s disposition to act prosocially and the feeling-oriented approach (e.g., Cialdini et al., 1987), stating that prosocial practices mainly rely on emotional regulation and positive emotional experiences, this study detected the mediating effect of TEI on the association between the dark triad and prosocial sustainability declined in terms of altruism and equity.

Hypotheses were formulated as follows:

H1: Machiavellianism, characterized by cynical behaviors and the inability to recognize emotions (Láng and Birkás, 2014), was found negatively associated with prosocial practices and TEI (Woodley and Allen, 2014; Miao et al., 2019); thus, TEI negatively mediates the interplay between Machiavellianism and both altruism and equity.
H2: Psychopathy, characterized by callous affect, erratic lifestyle, and antisocial behaviors (Neumann et al., 2007), was found negatively related to prosocial practices and TEI (Miao et al., 2019; Papageorgiou et al., 2020); thus, TEI negatively mediates the interplay between psychopathy and both altruism and equity.
H3: Although research on the association between narcissism and prosocial behaviors provides misleading results, narcissism mainly relies on a high desire for superiority that negatively impacts social relationships (Fanti and Henrich, 2015; Kauten and Barry, 2016). This led to an assumption that narcissism was negatively related to altruism and equity. Besides, grandiose narcissism was positively associated with TEI, whereas vulnerable narcissism was negatively related to TEI (Zajenkowski et al., 2018). Notably, although both grandiose and vulnerable narcissism are not separated into two factors by the Dirty Dozen used in this study, the scale captures both dimensions (Maples et al., 2014). Consequently, it was expected that (H3a) TEI negatively mediates the relationships between narcissism and prosociality, and (H3b) TEI positively mediates the relationships between narcissism and prosociality.

## Methods

### Participants and Procedure

A total of 129 healthy late adolescents (85 women; M_age_ = 19.96 years; SD_age_ = 1.05; range_age_ = 18–22 years) participated in the study. This study used a cross-sectional web-based survey design, and data were collected in September and October 2021. Using an online link distributed *via* different social media platforms, all participants were requested to electronically sign the informed consent, and complete the self-report measures and a short demographic questionnaire. Given the cross-sectional-based survey design, participants were selected from different regions of Italy, mostly from Abruzzo. The internal review board approved the study.

### Measures

The Dark Triad Dirty Dozen (Schimmenti et al., 2019) assesses Machiavellianism (e.g., *I tend to manipulate others to get my way*), psychoticism (e.g., *I tend to be unconcerned with the morality of my actions*), and narcissism (e.g., *I tend to seek prestige or status).* It consists of 12 items along a 5-point Likert-type scale (0, not at all; 4, very much). Cronbach’s α was 0.64 for psychopathy, 0.77 for Machiavellianism, and 0.82 for narcissism.

The Trait Emotional Intelligence Questionnaire—Short Version (TEIQue—SF; Di Fabio and Palazzeschi, 2011) evaluates TEI. The questionnaire was formed by 30 items along a 7-point Likert-type scale (1, completely disagree; 7, completely disagree) (e.g., *Expressing my emotions with words is not a problem for me*). In this study, the global TEI score was used and Cronbach’s α was 0.85.

The Altruistic Actions Scale (Corral-Verdugo et al., 2015) evaluates altruism as behaviors directed at helping others (e.g., visiting sick people and giving money to poor people). It consists of 10 items along a 4-point Likert-type scale (0, never; 3, always engage in such an action) (e.g., “*Provides some money to homeless*”). Cronbach’s α was 0.80.

The Equity Scale (Corral-Verdugo et al., 2015) taps behaviors directed at setting equal opportunities and treating conditions, regardless of gender and economic status. The scale is formed by seven items along a 5-point Likert-type scale (0, totally disagree; 4, totally agree) (e.g., “*Girls and boys have the same educational opportunities*”). Cronbach’s α was 0.61.

## Results

All statistical analyses were performed using IBM SPSS Statistic 24 for Windows. According to Hahs-Vaughn and Lomax (2020), data normality was tested by skewness and kurtosis values. Descriptive statistics are displayed in Table 1.

**TABLE 1:** Descriptive statistics for study variables.

Variable	*N*	Min	Max	*M*	SD	Skewness (std. error)	Kurtosis (std. error)
Machiavellianism	129	0.00	2.75	0.65	0.74	1.22 (0.21)	0.63 (0.42)
Psychoticism	129	0.00	3.00	0.81	0.73	0.92 (0.21)	0.55 (0.42)
Narcissism	129	0.00	3.50	1.01	0.88	0.79 (0.21)	−0.19(0.42)
TEI	129	2.00	6.81	5.06	1.09	−1.00(0.21)	0.72 (0.42)
Altruism	129	0.00	2.90	1.67	0.53	−0.06(0.21)	−0.24(0.42)
Equity	129	2.00	4.00	3.48	0.39	−1.18(0.21)	1.34 (0.42)

*TEI, Trait Emotional Intelligence.*

Spearman’s rho correlational analysis was computed to preliminarily evaluate the relationships involving the variables of interest (refer to Table 2), whereas to test the mediating role of TEI on the dark triad and prosocial behaviors, the PROCESS macro for SPSS (version 3.5; Hayes, 2017) was used. Specifically, we advanced six mediation models in which the three dimensions of the dark triad, namely, the focal predictors, altruism, and equity were the outcomes, and TEI was the mediator.

**TABLE 2:** Correlations among study variables.

	1	2	3	4	5	6
Machiavellianism (1)	1					
Psychoticism (2)	0.31**	1				
Narcissism (3)	0.60**	0.47**	1			
TEI (4)	−0.36**	−0.30**	−0.24**	1		
Altruism (5)	−0.37**	−0.22*	–0.15	0.41**	1	
Equity (6)	−0.29**	–0.12	−0.23**	0.27**	0.27**	1

N = 129. *p < 0.05, **p < 0.01 (two-tailed).

**FIGURE 1:**
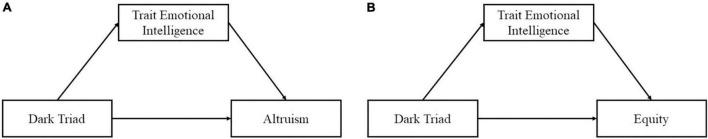
The mediating models advanced in this study, including the dark triad as the independent variable, Trait Emotional Intelligence (TEI) as the mediator, and altruism and equity as the outcomes. **(A)** TEI significantly mediated the relationship between Machiavellianism and altruism [indirect effect = −0.05, 95% CI (−0.1161, −0.0189); direct effect = −0.15, 95% CI (−0.2735, −0.0313); total effect = −0.21, 95% CI (−0.3324, −0.0897)], between psychoticism and altruism [indirect effect = −0.07, 95% CI (−0.1241, −0.0274); direct effect = −0.07, 95% CI (−0.2018, −0.0487); total effect = −0.14, 95% CI (−0.2730, −0.0231)], and between narcissism and altruism [indirect effect = −0.04, 95% CI (−0.0871, −0.0100); direct effect = −0.05, 95% CI (−0.1550, 0.0492); total effect = −0.09, 95% CI (−0.2016, 0.0090)]. **(B)** TEI significantly mediated the relationship between Machiavellianism and equity [indirect effect = −0.02, 95% CI (−0.0661, −0.0004); direct effect = −0.12, 95% CI (−0.2213, −0.0385); total effect = −0.15, 95% CI (−0.2448, −0.0669)], between psychoticism and equity [indirect effect = −0.03, 95% CI (−0.0780, −0.0024); direct effect = −0.09, 95% CI (−0.1924, −0.0041); total effect = −0.12, 95% CI (−0.2200, −0.0383)], and between narcissism and equity [indirect effect = −0.01, 95% CI (−0.0467, −0.0004); direct effect = −0.10, 95% CI (−0.1826, −0.0314); total effect = −0.09, 95% CI (−0.2005, −0.0504)].

Mediation analyses were examined through indirect effects with bootstrapped (samples = 5,000) SEs and bias-corrected 95% CIs. Considering altruism as the outcome, results showed that the indirect effects of Machiavellianism [*b* = −0.05, 95% CI (−0.1161, −0.0189)], psychoticism [*b* = −0.07, 95% CI (−0.1241, −0.0274)], and narcissism [*b* = −0.04, 95% CI (−0.0871, −0.0100)] on altruism through TEI were significant. Besides, the mediating analyses with equity as the outcome show that the indirect effects of Machiavellianism [*b* = −0.02, 95% CI (−0.0661, −0.0004)], psychoticism [*b* = −0.03, 95% CI (−0.0780, −0.0024)], and narcissism [*b* = −0.01, 95% CI (−0.0467, −0.0004)] on equity through TEI were also significant (refer to Figure 1).

## Discussion

Scientific research showed that adolescence involves increased perception, understanding, regulation, and functioning with emotions to achieve positive marks on behavior and personality, bringing youth to increase emotionality and prosociality, with consequences on various behaviors.

This study aims to investigate the involvement of TEI in association with the dark triad and social sustainability during late adolescence, a developmental stage characterized by a variety of changes, including dispositions toward the community (Zarrett and Eccles, 2006). Specifically, we advanced a mediation model, in which the dark triad (Machiavellianism, psychopathy, and narcissism) were the focal predictors, altruism and equity were the outcomes, and TEI was the mediator.

Results showed that Machiavellianism correlated negatively to both altruism and equitable behaviors; psychopathy correlated negatively only to altruism, whereas narcissism correlated negatively only to equitable behavior. This scenario revealed that the relationship between the dark triad and the different facets of prosociality is rather complex. Machiavellianism was confirmed as a dark trait fully oriented toward antisocial behaviors (Vernon et al., 2008; Muris et al., 2013); psychopathy as a disposition oriented specifically toward selfishness, affective callousness, and lack of empathy (Miller et al., 2011; White, 2014), and narcissism (presumably grandiose) as a trait negatively oriented toward equity.

Interestingly, TEI negatively mediated the relationships between the dark triad and both altruism and equitable behavior (H1, H2, H3b). In other words, lower levels in the dark triad were found to exhibit higher levels in TEI, which, in turn, positively impacted both altruism and equitable behavior. These findings suggest that prosociality results from the interaction between subclinical and malevolent personality traits, underpinned by the dark triad and positive emotion-related dispositions, which rely on TEI. In this vein, our results are in line with previous studies showing TEI as a protecting factor against the negative effects of the dark triad on different human behaviors, such as risk-taking and burnout (Grover and Furnham, 2021a,b). Specifically, TEI depicts a constellation of bright personality dispositions, including emotional management of others, emotion perception, social competence, and trait empathy, closely related to prosociality and cooperative practices (Petrides and Furnham, 2001; Petrides et al., 2007). This blend of positive dispositions and competencies, useful to increment people’s adaptability in different everyday life contexts (Fiorilli et al., 2019), could play an essential role in modeling negative dark triad dispositions into positive behavioral responses toward others.

To sum up, according to the feeling-oriented approach (e.g., Cialdini et al., 1987), stating that prosociality is closely related to emotionality, and personality theories on prosociality (e.g., Oda and Matsumoto-Oda, 2022), this study provides further empirical support on the protecting role of TEI against the malevolent and antisocial effects of the dark triad on prosociality. Notably, this mechanism assumes even more relevance during adolescence, a developmental stage that involves profound changes and represents a clear opportunity to increase, among others, socio-emotional abilities.

### Limitations and Future Directions

This study has at least two limitations worth noting. First, the researchers used a web-based survey design; thus, further studies should include performance tasks for measuring altruism and equity. Second, the dark triad was evaluated by a concise measure that did not highlight subfactors, such as primary and secondary psychoticism or grandiose and vulnerable narcissism. Thus, future research should consider more specific subdimensions of the dark triad to evaluate their differential effects on prosociality and TEI.

## Conclusion

Our findings enrich the knowledge about prosociality and provide a new perspective on the interaction of different facets of personality on social sustainability. Specifically, in this study, TEI results as a crucial factor against the effects of the dark triad on both altruism and equity, implying that late adolescents’ emotional perception and social effectiveness can be crucial in developing and maintaining positive attitudes and behaviors toward others. From an educational perspective, promoting emotional competencies, which support TEI, could reduce people’s antisocial dispositions and increase interest in prosocial practices and community efforts for social sustainability.

## Data Availability Statement

The raw data supporting the conclusions of this article will be made available by the authors, without undue reservation.

## Ethics Statement

The studies involving human participants were reviewed and approved by University of L’Aquila. The patients/participants provided their written informed consent to participate in this study.

## Author Contributions

MG performed conceptualization, methodology, formal analysis, investigation, data curation, writing the original draft, and writing review and editing. MP performed conceptualization, writing the original draft, and writing a review. SD’A performed conceptualization, supervision, resources, and writing review. All authors contributed to the article and approved the submitted version.

## Conflict of Interest

The authors declare that the research was conducted in the absence of any commercial or financial relationships that could be construed as a potential conflict of interest.

## Publisher’s Note

All claims expressed in this article are solely those of the authors and do not necessarily represent those of their affiliated organizations, or those of the publisher, the editors and the reviewers. Any product that may be evaluated in this article, or claim that may be made by its manufacturer, is not guaranteed or endorsed by the publisher.
